# HCV RNA viral load is independent from CD4 cell count and plasma HIV RNA viral load in immunocompetent HIV-HCV co-infected patients: a 3-years follow-up study

**DOI:** 10.1186/1742-6405-11-21

**Published:** 2014-07-29

**Authors:** Monica Basso, Marzia Franzetti, Renzo Scaggiante, Andrea Sattin, Carlo Mengoli, Mario Cruciani, Marta Fiscon, Giorgio Palù, Saverio Giuseppe Parisi

**Affiliations:** 1 Department of Molecular Medicine, University of Padova, Via Gabelli 63, 35100 Padova, Italy; 2 Center of Community & Medicine and HIV Outpatient Clinic, Verona, Italy

**Keywords:** HIV RNA, HCV RNA, Follow-up, Interleukin 28B CC allele, HCV genotype 1

## Abstract

**Background:**

HCV RNA viral load is an important predictor of sustained virological response and, recently, a significant correlation with liver fibrosis was described. We investigated on possible influence of clinical and viro-immunological variables on HCV viral load in HIV-HCV co-infected patients over a study time of three years (2009-2012).

**Methods:**

We retrospectively enrolled 98 adult patients with a diagnosis of chronic HIV infection in 2009, a diagnosis of chronic HCV infection with a detectable plasma HCV RNA in 2009 and 2012, HCV therapy-naïve or with failed and stopped antiviral treatment before June 2008. The following variables were recorded: age, gender, HCV genotype, IL28B rs12979860 CC genotype, HCV treatment status, advanced liver fibrosis diagnosis, antiretroviral therapy, CD4+ cell count, HCV viral load, HIV RNA (plasma HIV-1 RNA levels were measured from blood samples every three months at least). The correlation was established using linear regression analysis, analysis of variance and Fisher’s exact test. Comparisons between groups were performed using Fisher’s exact test, the independent samples t-test and the t-test for paired data, as appropriate, for continuous variables. A mixed mode (ME) maximum likelihood linear regression model was constructed to evaluate the dependence of HCV viral load.

**Results:**

HCV RNA levels did not change significantly from 2009 to 2012 (from 3924650 ± 5320177 IU/ml to 3085128 ± 3372347 IU/ml, *p* = 0.13); the CD4+ count increased significantly (from a mean of 576 to a mean of 654, *p* = 0.003). Using linear regression, a positive correlation was observed for HCV load and genotype 1 (*p* = 0.002), nonresponder status (*p* = 0.04) and with interleukin 28B CC allele (*p* = 0.05). Other studied covariates failed to reach a significant correlation.

**Conclusions:**

The HCV RNA load, a known pretreatment predictor of response to antiviral therapy, was independent of the two main parameters of HIV disease, plasma HIV RNA and CD4 cell count, over an observation time of 3 years in patients with recovered or spontaneously maintained immunocompetence.

## Background

Hepatitis C virus (HCV) is the cause of a significant proportion of cases of chronic liver disease. In human immunodeficiency virus (HIV)-HCV co-infected patients effectively treated with HAART (highly active antiretroviral therapy), HCV-related morbidity and mortality has increased alarmingly in recent years [1]. The treatment of HCV disease with pegylated interferon and ribavirin is challenging in HIV-positive patients, but reductions in liver-related and liver-independent mortality have been demonstrated in patients who achieved sustained virological response (SVR) [2]. The efficacy of therapy with pegylated interferon and ribavirin is lower than in mono-infected patients; the SVR rates are 38% in naïve patients and 28% with retreatment [3,4]. Ideally, the timing of HIV and HCV therapies must be individualized and tailored based on personal patient clinical characteristics, which can change with time; this approach is especially recommended for HIV patients with HCV genotype 1 infection, who may be candidates for antiviral treatment, including direct-acting antiviral drugs (DAAs) [5].

A low pretreatment HCV RNA load is a known positive factor of SVR [6]. The recently published European AIDS Clinical Society Guidelines stated that a low pretreatment HCV RNA value is 400,000-600,000 IU/mL [7]. A pretreatment HCV RNA viral load < 600,000 IU/ml is significantly associated with SVR in co-infected patients treated with pegylated interferon and ribavirin (p < 0.001) [8], even in subjects with compensated cirrhosis (*p* = 0.01) [9]; conversely, a value higher than this threshold is a predicting factor for relapse (*p* = 0.02) in the study by Rivero-Juarez et al. [10]. The HCV RNA viral load is thought to play a role in fields other than antiviral treatment; Kirk et al. [11] reported a significant correlation between liver fibrosis and HCV RNA load (*p* < 0.001) in a cohort of 1176 HCV-positive subjects (34% with HIV coinfection).

Studies on clinical and viro-immunological correlates of HCV RNA load in HIV patients reported conflicting results. In a cross-sectional study by Collazos et al. [12], HCV RNA load was lower in subjects with undetectable HIV viremia (6.17 log_10_ IU/mL versus 6.08 log_10_ IU/mL, *p* = 0.03), whereas Grint et al. [13] demonstrated that only ongoing HAART is a predictive factor of HCV viral load stability over time, with an increase of 2.6% per year compared to 27.6% in untreated subjects (*p* = 0.009). Plasma HIV viremia is a dynamic variable; patients taking antiretroviral therapy (ART) may develop virological breakthrough or experience side effects leading to treatment interruption, and untreated subjects may start antiretroviral drugs.

In this work, we investigate whether HCV viral load (quantified at the beginning and at the end of the study) is associated with the demographic, clinical and virological characteristics of HIV-HCV-co-infected patients. We performed HCV RNA analysis, both as a continuous value and as a discrete variable (low HCV RNA when viral load was lower than 600,000 IU/ml).

## Results

**Figure 1:**
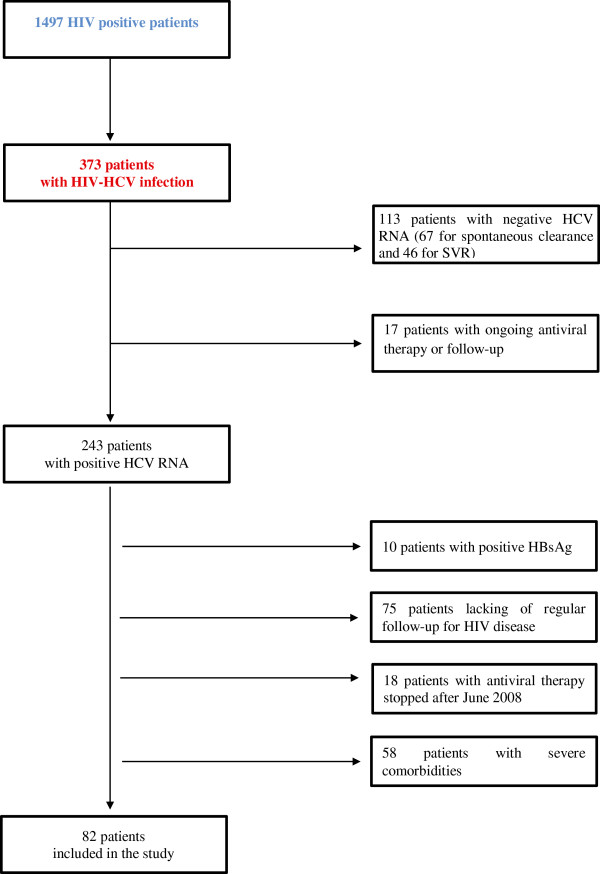
Flow chart describing the selection of the study patients.

A total of 1497 patients with HIV infection were followed at the Infectious Disease Unit of Padova Hospital in Italy in 2012. In 373 subjects, a diagnosis of HCV co-infection was reported. A complete description of the selection process is summarized in Figure 1.

**Figure 2:**
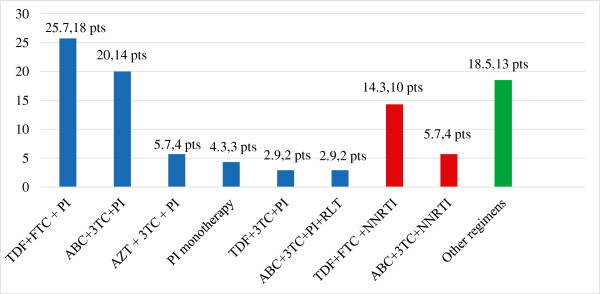
**Antiretroviral drug regimens of the 73 co-infected patients treated in 2012 (data available for 70/73 patients, 95.6%): for each drug combination are reported both percentage respect to the number of treated patients and absolute number.** Legend: ABC = abacavir; AZT = zidovudine; FTC = emtricitabine; MVC = maraviroc; NNRTI = non-nucleoside reverse transcriptase inhibitors; PI = protease inhibitor; RLT = raltegravir; TDF = tenofovir; 3TC = lamivudine.

The majority of the patients were Caucasian (80/82, 97.6%). Genotype 3 infection was the most frequent non-type 1 infection (20 patients, 24.4%), 12 patients (14,6%) had genotype 4 and only 2 subjects had genotype 2 (2.4%). The diagnosis of advanced liver fibrosis was based on clinical and not invasive evaluation in most patients; Overall a liver biopsy was performed in only 10 patients (12.2%). The diagnosis of advanced liver fibrosis was performed by invasive evaluation only in one patient, liver stiffness data were available for 8 out the 19 patients (42.1%). Among the 17 patients who did not achieve an SVR, 8 (47%) had an HCV genotype 1 infection. The baseline characteristics of the included patients are described in Table 1.At the end of the study period, 73 patients were on ART. In 63 subjects (86.3%), no treatment interruption was reported. Among subjects who never stopped ART, successful HIV viremia suppression was observed in 37/63 (58.7%) A total of 14/82 (17.1%) patients showed HIV RNA suppression in only one of the two periods included in the IFU. Among these 14 patients, 9 of the 11 subjects with ongoing ART achieved successful HIV RNA suppression during FFU; thus, the overall percentage of treated subjects with successful suppression at the end of the study period was .46/63 (73%). A detailed description of the 2012 antiviral regimen is reported in Figure 2.

**Table 1:** Demographic, clinical and virological features of the 82 HIV-HCV co-infected patients who fulfilled the inclusion criteria

**Variables**	**Results**
Age (years)^1^	48 (±6)
Male, n (%)^2^	62 (75.6)
HCV Genotype 1, n (%)^2^	48 (58.5)
Previous HCV therapy, n (%)^2^	17 (20.7)
Advanced liver fibrosis, n (%)^2^	19 (23.2)
CD4 cell count at T1^1^ (cells/mm^3^)	576 (±280)
CD4 cell count at T2^1^ (cells/mm^3^)	654 (±351)
HCV RNA at T1^1^ (IU/ml)	3924650 (±5320177)
HCV RNA at T2^1^ (IU/ml)	3085128 (±3372347)
Ongoing ART at T2^2^, n (%)	73 (89)
Clean IFU, n (%)^2^	39 (47.6)
Clean FFU^5^, n (%)^2^	57 (69.5)
Discordant HIV RNA suppression at IFU^4^, n (%)	14 (17.1)
IL 28B CC genotype, n (%)^2^	20 (31.7)

^1^mean and SD; ^2^absolute number and percentage respect to all included patients.

ART: Anti Retroviral Therapy; Clean IFU: HIV viremia undetectable during initial follow-up (0-24 months from T1), regardless of being on ART; Clean FFU: HIV viremia undetectable during final follow-up (24-36 months from T1), regardless of being on ART.

Data on IL28B genotype were available for the 63 patients (76.8%).

The CD4+ count increased significantly from T1 to T2, with a mean increase of 78 cells/mm3 (from 576 to 654 cells/mm3, *p* = 0.003), and the T2 value correlated positively with the T1 count (*p* = 0.0001). Undetectable plasma HIV viremia at IFU predicted HIV RNA suppression at FFU (*p* = 0.0001) and was positively correlated with the CD4+ cell count at T2 (*p* = 0.03). Undetectable plasma HIV viremia at IFU was correlated with the CD4+ cell count at T1 (*p* = 0.04). The HCV RNA level did not change significantly during the observation period (*p* = 0.71). In 11 (13.4%) patients a low HCV RNA viral load was reported both at T1 and T2 and in 22 (26.8%) subjects only at T1. Low HCV RNA viral load at T1 had a positive association with low HCV RNA at T2 (*p* = 0.001) and a negative correlation with the CD4+ cell count at T2 (*p* = 0.01).

The ME linear regression revealed significant positive correlations between log_10_ HCV RNA and HCV genotype 1 (*p* = 0.002), previous failed HCV treatment (*p* =0.04) and harboring the IL28B CC allele (*p* = 0.05). All other explanatory covariates failed to reach significant P values, including the successful suppression of HIV viremia or persistent ongoing viral replication (irrespective of the study period analyzed) and ongoing ART at the end of follow-up (Table 2). The predicted HCV viremia margins after ME linear regression were calculated, including the three significant predictors of high HCV viral load (Table 3).

**Table 2:** **Mixed mode (ME) maximum likelihood linear regression model test with log**_**10 **_**HCV RNA as dependent variable**

**Log**_**10 **_**HCV RNA**	**Coef.**	**Std. Err.**	**z**	**P**	**95% Conf.**	**Interval**
Age	0.011	0.021	0.50	0.614	-0.030	0.051
Gender	0.068	0.194	0.35	0.727	-0.313	0.448
HCV genotype 1	0.543	0.178	3.04	0.002	0.194	0.893
Advanced liver fibrosis	-0.183	0.205	-0.89	0.372	-0.585	0.219
ART	-0.175	0.284	-0.62	0.537	-0.731	0.381
CD4 cell count/mm3	0.000	0.000	-0.21	0.831	-0.001	0.000
Previous HCV therapy	0.378	0.185	2.05	0.041	0.016	0.740
IL28B CC genotype	0.331	0.168	1.96	0.050	0.001	0.660
_cons	5.229	1.047	5.00	0.000	3.178	7.281

Log likelihood = -95.223521, Wald chi^2^(8) = 18.96, P = 0.0151.

ART: Anti Retroviral Therapy; Clean IFU: HIV viremia undetectable follow-up, regardless of being on ART.

**Table 3:** Predicted HCV viremia, margins after ME linear regression

**HCV genotype 1**	**Previous HCV therapy**	**IL28B CC**	**Margin (IU/ml)**	**95% Conf. interval (IU/ml)**	
No	No	No	5.16x10^5^	2.55x10^5^	1.04x10^6^
No	No	Yes	9.13x10^5^	3.90x10^5^	2.14x10^6^
No	Yes	No	1.02x10^6^	4.34x10^5^	2.42x10^6^
No	Yes	Yes	2.95x10^6^	8.78x10^5^	9.94x10^6^
Yes	No	No	1.66x10^6^	9.61x10^5^	2.87x10^6^
Yes	No	Yes	3.36x10^6^	1.68x10^6^	6.71x10^6^
Yes	Yes	No	3.86x10^6^	1.70x10^6^	8.74x10^6^
Yes	Yes	Yes	7.46Ex10^6^	3.08x10^6^	1.81x10^7^

Three predictors were included, HCV genotype 1, previous anti HCV therapy, and IL28=CC, in all combinations.

## Discussion

The objective of this longitudinal study was to analyze the possible influences of clinical and virological parameters on plasma HCV replication evaluated at the beginning and at the end of a time interval of 3 years. Our data indicate that age, gender, a diagnosis of advanced liver fibrosis, CD4+ cell count and successful HIV therapy, leading to undetectable plasma virema, failed to exhibit significant effects on HCV RNA levels.

Furthermore, we focused our interest on the characteristics of HIV-HCV-co-infected patients with persistent serum HCV RNA levels lower than 600,000 IU/ml. Co-infected patients with persistent low HCV RNA levels are typically infected with non-genotype 1 viruses and represent a small proportion (13.4%) of our population of HIV-HCV-co-infected subjects. However, when only baseline data are considered, the proportion of co-infected patients increased to 26.8%, a figure comparable to the 25% reported in the study by Rallon et al. [14] in a cohort of 196 co-infected patients. The association between HCV genotype 1 and the IL28B C allele with higher HCV RNA values is described in HIV-HCV-co-infected patients [11,15]; these findings demonstrated that no unintended selection bias occurred.

Barreiro et al. [16] stated that subjects with HIV-HCV co-infection are more likely than HIV-negative subjects to experience variations in HCV RNA load greater than 0.5 log over a mean period of 43 months. The number of patients surveyed was greater than that in our study (644 versus 82), but the proportion of patients on ART was similar to ours (82% and 86.7%, respectively). Only two HCV RNA controls were available in our study because there was no clinical indication to prompt more frequent measurements. Nevertheless, increases of 0.5 log IU/ml of both the baseline and the final mean HCV RNA values for the patients categorized as having low-level viremia would correspond to values below 800,000 IU/ml, the threshold accepted as “low” [17]; therefore, our patients maintained their virological classification throughout the study period.

Most included patients (76.8%) were on ART, but a persistent undetectable HIV RNA load with a cut-off of 50 copies/ml was observed in only 58.7% of patients. At a comparable end point (144 weeks of follow-up), the cross-study analysis by Hua et al. [18] on 279 HIV-HCV-naïve co-infected patients described a rate of virological failure (defined as plasma HIV viremia > 200 copies/ml) of 10.5% (4 patients out of 38 evaluable patients). Of note, HCV status was defined only serologically and not by the presence of detectable HCV RNA, and HBsAg-positive patients were included, albeit the percentage was low (4.3%). ART efficacy was not the purpose of our work; nevertheless, we can report an interesting finding on the long-term efficacy in a small but homogeneous cohort of all HCV patients positive for HCV RNA, with no HBV coinfection and of Caucasian ethnicity who were treated according to the updated HIV guidelines.

Grint et al. [13] described HCV viral load changes in 1541 patients selected from the EuroSIDA study cohort: the median follow-up was five years, and the only factor able to stabilize HCV viral load was ART. In this study, 1148 subjects (74.5%) were on ART at baseline, with undetectable HIV RNA levels in 657 patients (57.2%); 393 subjects were not taking ART (88.2% with a detectable HIV RNA). Despite this virological picture, the absence or presence of detectable plasma HIV viremia has no significant influence on HCV RNA viral load. Successful ART and undetectable HIV plasma viremia are obviously strictly related if we exclude long-term non-progressing patients. We considered our patients to be responders to therapy when viral replication was lower than 50 copies/ml (400 copies/ml in the study by Grint et al.); however, such a threshold identifies a clinically successful suppression but does not indicate definitively absent plasma HIV replicative activity. There is mounting evidence on the prognostic role of HIV residual viremia (2.5-50 copies/ml) [19], and we cannot exclude the possibility that residual viremia may influence HCV RNA viral load, as could greater HIV burden. No correlation was observed between CD4+ cell count and HCV RNA values as previously described [12]; CD4+ cell count increased from T1 to T2 (mean increase of 78 cells/mm^3^, *p* = 0.003). The baseline immunocompetence in our enrolled patients was greater than that reported for subjects in the study by Grint et al. 576 cells/mm3 versus 340 cells/mm^3^ in the subjects on ART [13].

Interestingly, low HCV RNA viral load at T1 was negatively correlated with CD4+ cell count at T2 (*p* = 0.01). This finding does not contradict our other data because it was described in a selected population of patients: those with low HCV RNA viral load. This report is the first to describe this correlation, and only speculative hypotheses can be made, including the inverse correlation between CD4 cell apoptosis and serum HCV RNA [20].

There is a growing evidence of a low rate of prescription of antiviral treatment in co-infected patients. The study by Ioannou et al. [21] reported that only 994 out of 5999 (18%) HIV-HCV-co-infected patients assisted by the Veterans Affairs healthcare system received an interferon-based regimen. This percentage rises to 25.3% in the EuroSIDA cohort but is accompanied by a reduction in treatment incidence in 2009 [22]. The proper timing at which to begin pegylated interferon and ribavirin treatment, associated with DAA when indicated, must take HIV disease status into account, but such coordination is not an easy task. Concomitant ART may be a risk factor for the development of side effects even in the case of therapy with pegylated interferon and ribavirin without DAA; However, a CD4+ cell number greater than 450/mmc predicts SVR in co-infected patients with HCV genotype 1 [23,24].

## Conclusions

The strengths of this study can help treating physicians to tailor the beginning of HCV disease treatment because we demonstrated that HCV RNA value, a known pretreatrment predictor of response to antiviral therapy, remain independent of the two main parameters of HIV disease, plasma HIV RNA detectability and CD4+ cell count, over an observation time of 3 years and in patients with recovered or spontaneously maintained immunocompetence.

## Methods

### HIV-HCV co-infected patients

We retrospectively enrolled patients into the study on the basis of the following eligibility criteria: 1) older than 18 years; 2) diagnosis of chronic HIV infection in 2009; 3) diagnosis of chronic HCV infection with a detectable plasma HCV RNA in 2009 and 2012; 4) hepatitis B serum antigen (HBsAg)-negative in 2009 and 2012; 5) HCV therapy-naïve or with failed and stopped antiviral treatment before June 2008; 6) regular viro-immunological follow-up of HIV disease from 2009 to 2012; 7) no pregnancy at the time of enrollment and throughout the time of the study; and 8) no severe comorbidities.

Severe comorbidities were defined as any pathological condition reported to increase HCV RNA load (i.e., intravenous drug use or alcohol abuse).

The enrolled subjects gave informed consent for all procedures and for the use of their blinded data for scientific evaluation and publication. This study was conducted in accordance with the Helsinki Declaration and local legislation and was approved by the local Ethics Committee.

Throughout the study period, the patients were untreated for HIV infection or received antiretroviral treatment according to the current international guidelines. Decisions on HIV therapy were at the discretion of the treating physician; there were no restrictions on CD4+ cell counts in 2009 and 2012.

### Study time

**Figure 3:**
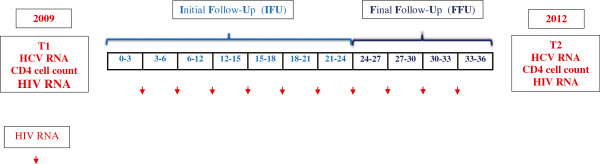
**Study design overview.** Initial Follow-Up (IFU) started at T1 (in 2009) and lasted 2 years, Final Follow-Up (FFU) started at the end of IFU, lasted one year and finished at T2, in 2012.

Two time periods were identified: Initial Follow-Up (IFU) and Final Follow-Up (FFU). IFU started at T1 (in 2009) and lasted 2 years. The end of IFU coincided with the start of FFU, a one-year period closer to the end of the study, which finished at T2, in 2012 (Figure 3).

### Study of HIV infection

Plasma HIV-1 RNA levels were measured from blood samples at all visits (every three months at least). The lower limit of detection of the commercial test changed over the course of the study from 20 to 40 copies/mL, but we considered plasma HIV viremia as undetectable when the viral load was below the limit of 50 copies/ml [25].

Patients were classified as having undetectable HIV viremia if no plasma value exceeded 50 copies/ml; only a single viral blip throughout every year (an increase in the HIV RNA plasma viral load above 50 copies/ml but lower than 500 copies/ml and followed by undetectable HIV RNA) was tolerated [26].

HIV viremia was classified as detectable if any other result occurred. The detectability of HIV viremia was analyzed for each period of 12 months and was categorized as successful suppression or no suppression. Patients who reported different results in the HIV viremia control during the two 12-month periods included in IFU were considered to exhibit no HIV RNA suppression, with the aim of clearly identifying the cohort of HIV-HCV-co-infected patients with persistent plasma HIV RNA levels < 50 copies/ml.

Patients were classified as taking ART in 2012 when the HCV RNA was measured if the treatment had been ongoing for at least three months.

### Study of HCV infection

A diagnosis of advanced liver fibrosis was made when a liver biopsy revealed a result corresponding to Metavir scores F3 and F4 or when liver stiffness measured by a transient elastography by FibroScan (Echosens, Paris, France) had a value higher than 9.5 Kpa [27]. If these data were not available the estimation of hepatic fibrosis was made on clinical, laboratory and ultrasound data as described by Pineda et al. [28]. Therapy with pegylated interferon and ribavirin was not started during the study time because of the lack of urgent indication or due to patient refusal. Previously failed antiviral treatment included therapy with interferon (standard or pegylated) as monotherapy or with ribavirin according to the current international guidelines at the moment of treatment start. Any virological response different from SVR was classified as no response to antiviral treatment.

### Virological methods

HCV RNA genotypes were determined using the VERSANT® HCV genotype 2.0 assay (INNOLiPA, Innogenetics, Belgium). HIV and HCV plasma viral loads (copies/ml) were evaluated using different platforms: until July 2011 with the COBAS TaqMan assay (Roche, Basel, Switzerland) and thereafter using the Abbott RealTime assay (Abbott RT), (Abbott Molecular Inc., Des Plaines, IL).

Interleukin (IL)28B polymorphism genotyping at single-nucleotide polymorphism locus rs12979860 was conducted using a validated in-house method. The IL28B allele was classified as CC or not CC (including CT and TT) for statistical analysis.

### Statistical analysis

The following variables were recorded: age, gender, HCV genotype 1 (binary, HCV genotype 1 versus non-genotype 1), IL28B CC genotype (binary, CC genotype versus CT/TT genotypes), previously failed anti-HCV treatment versus naïve to antiviral therapy (binary, yes versus no), advanced liver fibrosis (binary, presence versus absence of advanced liver fibrosis), antiretroviral therapy (binary, ongoing versus no antiretroviral therapy ongoing at T2), CD4+ cell count at T1 and at T2, HCV viral load at T1 and at T2, IFU HIV RNA levels (binary, bad versus good HIV RNA suppression) and final follow-up HIV RNA levels (binary, bad versus good HIV RNA suppression).

Moreover, the following derived variables were obtained: log_10_ HCV RNA at T1 and log_10_ HCV RNA at T2 after log_10_ transformation of T1 HCV RNA and T2 HCV RNA, respectively.

The variables were submitted to pairwise correlation analysis to outline the underlying pattern in the evaluated population. The correlation was established using linear regression analysis, analysis of variance (ANOVA) and Fisher’s exact test. Comparisons between groups were performed using Fisher’s exact test, the independent samples t-test and the t-test for paired data, as appropriate, for continuous variables.

A mixed mode (ME) maximum likelihood linear regression model was constructed. The following time-varying covariates were obtained: CD4+ cell count, logHCV viremia and successful HIV viremia suppression. The dependence of HCV viral load on a set of time-invariant (age, gender, HCV genotype 1, advanced liver fibrosis, previous failed HCV therapy) and time-varying covariates was established. The limit of significance for all analyses was established at *p* < 0.05.

### Ethical approval

The local government, which was represented by the Veneto Regional Health Authority, approved the study and provided funding (Regional Government Decrees 3643/2004 and 3499/2008). This study was conducted in accordance with the Helsinki Declaration and local legislation (Ethics Committee of Padova University Hospital, prot. 2606-12P).

## Abbreviations

ART: Antiretroviral therapy; DAAs: Direct-acting antiviral drugs; FFU: Final follow-up; HAART: Highly active antiretroviral therapy; HCV: Hepatitis C virus; HIV: Human immunodeficiency virus; IFU: Initial follow-up; SVR: Sustained virological response.

## Competing interests

The authors declare that they have no competing interests.

## Authors’ contributions

MB collected the data, interpreted the findings, and wrote the paper; MF managed the patients and interpret the findings; RS managed the patients and wrote the paper; AS managed the patients and helped to interpret the findings; CM interpreted the data and performed the statistical analysis; MC helped to interpret the findings and wrote the paper; MF helped to interpret the findings and write the paper; GP helped to design the study and write the paper; SGP designed and coordinated the study, interpreted the findings, and wrote the paper. All authors read and approved the final manuscript.
